# In-fibre particle manipulation and device assembly via laser induced thermocapillary convection

**DOI:** 10.1038/s41467-019-13207-0

**Published:** 2019-11-15

**Authors:** Jing Zhang, Zhe Wang, Zhixun Wang, Ting Zhang, Lei Wei

**Affiliations:** 1 School of Electrical and Electronic Engineering, Nanyang Technological University, 50 Nanyang Avenue, Singapore, 639798 Singapore; 2 Institute of Engineering Thermophysics, Chinese Academy of Sciences, 100190 Beijing, China

**Keywords:** Engineering, Materials for devices, Applied optics, Optical materials and structures, Electronics, photonics and device physics

## Abstract

The ability to manipulate in-fibre particles is of technological and scientific significance, yet particle manipulation inside solid environment remains fundamentally challenging. Here we show an accurately controlled, non-contact, size- and material-independent method for manipulating in-fibre particles based on laser-induced thermocapillary convection. The laser liquefaction process transforms the fibre from a solid media into an ideal fluid environment and triggers the in-fibre thermocapillary convection. In-fibre particles, with diameter from submicron to hundreds of microns, can be migrated toward the designated position. The number of particles being migrated, the particle migration velocity and direction can be precisely controlled. As a proof-of-concept, the laser-induced flow currents lead to the migration-to-contact of dislocated in-fibre p- and n-type semiconductor particles and the forming of dual-particle p-n homo- and heterojunction directly in a fibre. This approach not only enables in-fibre device assembly to achieve multi-component fibre devices, but also provide fundamental insight for in-solid particle manipulation.

## Abstract (web summary)

Particle manipulation inside a fibre is challenging yet meaningful to achieve for fibre devices. Here, the authors demonstrate a method to manipulate in-fibre particles via laser induced thermocapillary convection, enabling the formation of semiconductor junctions directly in a fibre.

## Introduction

The recent development of multi-material fibres and techniques for in-fibre materials engineering opens a new frontier for developing fibres with more advanced functionalities other than transmitting lights. Thermally drawn fibres from macroscopic preforms can comprise a wide range of materials including semiconductors, metals, polymers or organic materials^1–4^. In particular, micro-sized fibres can be further woven into large-area flexible fabrics, generating several novel technologies for wearable or implantable applications such as optical and chemical sensing^5–7^, piezoelectric and triboelectric actuation^8–12^, and biological signals detection and recording^13,14^. As compared to traditional fibres with typical cylindrical cladding-core structures, multi-material enabled functional fibres require higher complexities in the fibres’ internal structures. Methods such as optical treatment^15^, chemical treatment^16^, mechanical treatment^17^ and thermal treatment^15,18–24^ that capably define fibres’ internal structures as well as modify materials’ properties have been investigated. For example, in the case of thermally induced capillary instability in fibre, varisized spherical particles of different functional materials can be generated internally based on the selective break-up of continuous fibre core, providing numbers of optoelectrical components to be achieved in the multi-material fibre^25–27^. However, as the fibre cladding materials are normally in the solid phase, the existing methods can only generate periodic in-fibre particles all at fixed positions with monotonous architectures that are frozen in situ upon cooling, which significantly limits the degrees of freedom for developing in-fibre functional devices within the solid media. Notably, methods to precisely manipulate small subjects such as optical trapping^28,29^, optoelectronic tweezers^30,31^, far-field and near-field trapping^32–36^ have been well studied and applied maturely for samples in fluidic media. However, challenges arise to directly apply these techniques into fibre technologies whose functional devices are embedded in the solid cladding that are not accessible in common fluidic media. Therefore, to achieve precisely designed in-fibre multi-material device with spatially defined functional architectures and to across the gap of materials, methods to manipulate and migrate of these generated in-fibre subjects are greatly needed.

Here we present an approach to accurately manipulate and migrate in-fibre particles for constructing novel multi-material functional fibres. This approach combines the advantages of laser induced Plateau-Rayleigh instability for generating particles made by different materials and the same-laser induced thermocapillary convection for re-locating the spatial positions of these particles, thus the assembly of complex in-fibre device becomes much practical. Because of the thermal liquefaction process induced by the CO_2_ laser heating, the solid fibre cladding can be transferred into an ideal fluidic media, enabling the thermocapillary convection manipulation to move in-fibre particles to any designed positions. The thermocapillary effect studied in this paper is a common physical phenomenon, it is observed in daily life which occurs when there is a gradient of surface tension in liquid^37,38^. It has long been used in microfluidics applications as a non-contact, stable, size- and material-independent transport mechanism for trapping, filtering, pumping and migrating varisized particles^39–41^. To develop this phenomenon in-fibre for particles manipulation, we use the CO_2_ laser to heat the solid cladding, which introduces a gradient of surface tension within the fluidised fibre, therefore particles migrate away from their original positions by the gradient forces, determined by the laser spot. Various factors that determine this thermocapillary process are investigated theoretically and experimentally. Results suggest that the moving directions, velocities and the number of particles being manipulated can be accurately controlled through the presented approach. Furthermore, this approach is validated by successfully forming in-fibre dual-particle semiconductor homo- and heterojunction.

## Results

### In-fibre thermocapillary convection and particle migration

**Fig. 1:**
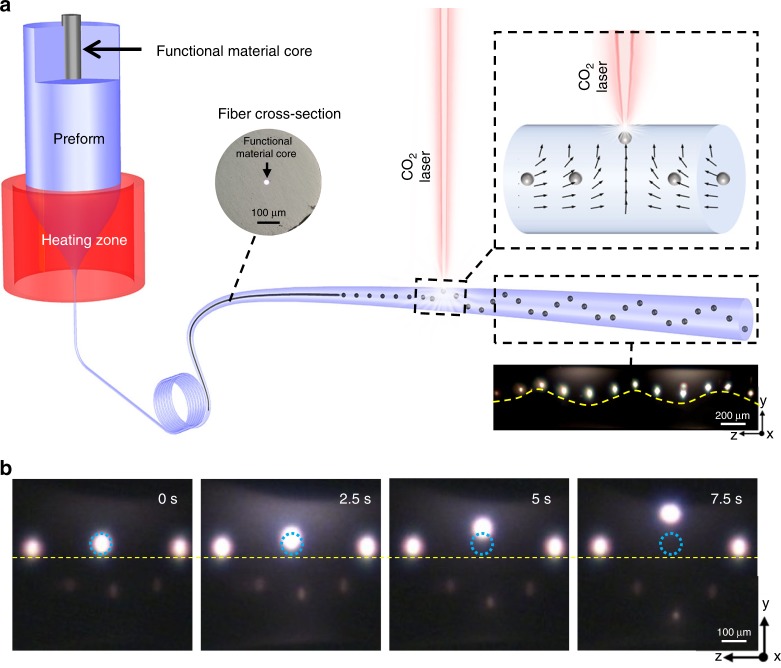
CO_2_ laser induced thermocapillary convection for in-fibre particle manipulation and structure assembly. **a** Schematic of the multimaterial fibre thermal drawing process and in-fibre structure assembly based on CO_2_ laser induced thermocapillary convection. The inset on the top right corner shows the velocity profiles of the convection flows which drag the particles. The inset on the right bottom corner shows a reconstructed germanium-in-silica fibre with the germanium particles on a sinusoidal distribution. **b** Snapshots of an in-fibre single particle with a diameter of 40 μm guided and migrated by the CO_2_ laser power at different time-points. At the beginning (*t* = 0 s), the particle is located at the centre of the fibre cladding. Starting from the laser switching on, the particle is dragged away from fibre centre and migrated towards the cladding surface (*t* = 7.5 s) where the laser is focused on. Blue circle indicates the original position when particle is formed

Functional material-core glass-cladding fibre is firstly drawn from a macroscopic preform (See Methods and Supplementary Note 2 for details), and the fabricated fibre is fed into a CO_2_ laser to form in-fibre particles. With the laser power being increased, solid fibre cladding and core materials are transferred into liquid phase. According to the capillary instability theory, when a liquid cylinder (fibre core) is surrounded by another liquid (fibre cladding), a perturbation wave occurs on the interface due to the competition of surface tension and viscoelastic force between two materials. The perturbation wave further grows and breaks the fibre core into a chain of spheres (Fig. 1a, see Methods for details). Next, as the laser continuously heating, the generated spherical particles start to drift away from their original positions and float upwards to the laser heating point (Fig. 1a). Such a relocation of the in-fibre particles is due to the hydrodynamic fluid convection induced by the laser heating treatment.

To understand the convection flows and particle migrations in the fibre, we use microfluidic dimensionless numbers to analyze and determine the type of convection^42^. There are two types of flow participating this toroidal convection: the nature convection flow and the thermocapillary flow driven by the surface tension force, viscous force and the buoyancy force. In the case that the fibre radius is around ~250 µm, the natural convection can be ignored^40^. Furthermore, our calculations suggest that the Marangoni Number (Ma) is 10^4^ larger than the Rayleigh Number (Ra), indicating that the Marangoni convection is the dominant mechanism in this situation (Details of calculation can be found in Supplementary Note 1).

The thermocapillary effect, also known as the thermal Marangoni effect, is a natural fluid motion driven by the temperature-dependent surface tension gradient. It is well understood in macroscopic engineering applications, such as welding and metal processing^43–45^. As this effect also exists in microscale fluids, thermocapillary convection has further pathed a new way into mesoscale and microscale microfluidic and optofluidic applications, such as transportation and mixing of microparticles and formation of self-organised structures^46–48^. Since the liquid with a higher surface tension would pull subjects towards the liquid with lower surface tension, the gradient in surface tension force results in a mass transfer. Observations suggest that the thermocapillary convection is centred at the heating spot. Thus, the inner liquid currents move parallel to the heating spot and direct towards the liquid–air interface while the liquid at the interface drifts away from the heating points, forming the convection (Fig. 1a, dash box). The convection flow will drive the particles suspended in liquid to overcome the Stokes’ drag force and start to migrate towards the heating spot, resulting in a promising approach for particle trapping and manipulation^49^. To accurately control the thermocapillary convection for manipulating in-fibre particles, we introduce the thermocapillary convection into the multi-material fibre by using a CO_2_ laser, which can provide a powerful heating source with an extremely stable power and precisely controlled spot sizes. In this case, laser heating liquidises a small portion of fibre cladding, establishes a temperature-depended surface tension gradient, and induces a locally confined thermocapillary convection. The stable fluidic convection within the liquidised cladding carries the embedded particles to leave their original position and move towards the heating point (Fig. 1a, dash box). By directing the position of laser spot, the location and direction of fluid convection can be well controlled, which indicates that this method can be applied to move particles at any position along the fibre on demand.

Furthermore, according to the definition of Marangoni number, active range and flow velocity are the two key parameters for the thermocapillary convection. They are determined by the following physical attributes: heating temperature, temperature gradient, temperature coefficient of surface tension, and material viscosity. Therefore, precise control of these characters provides a promising way to accurately control the in-fibre thermocapillary convection, thus precisely manipulating the embedded particles for in-fibre fabrication becomes possible.

Notably, the manipulation of objects is driven by the flowing motions of medium without any interactions between objects, lights and fibre materials, providing several distinguished merits. First, this approach is universal. As the movements of the in-fibre particles are driven by the fibre cladding material’s motion, which is universal for all subjects in the flow, there is no limitation over the particle sizes, shapes and materials, whatever they are conductive, dielectric or semiconductors for performing this method. Second, the laser heating generated toroidal shaped convection allows the migration of the in-fibre heavy objects as long as the laser provides sufficient energy, making it not only suitable for sub-micron but also mesoscale particles. Lastly, this approach is a non-contact method, which avoids contaminations or mechanical damages of samples.

### Challenges in precisely control of particle manipulation

To start the thermal treatment, the laser beam is focused on the selected zone of the fibre, as shown in Fig. 1a. By absorbing laser energy, the fibre cladding material reaches its glass transition temperature and transforms from solid phase to liquid phase, which makes the fibre from a solid media that is not suitable for particles manipulation into an ideal fluid environment for the in-fibre thermocapillary convection to take place.

We firstly verify this method using a silica fibre embedded with germanium particles, which are formatted with the same diameter and distributed in a stable period. The CO_2_ laser induces a heating field on the fibre and establishes a stable gradient of temperature-dependent surface tension in order to trigger the thermocapillary convection. Figure 1b shows the manipulation of a single particle in the fibre. In this experiment, the CO_2_ laser beam with a laser power of 4 W and a spot size of 500 µm is used to liquidise a selected zone of fibre with spherical particles with a diameter of 30 µm and distribution period of 125 µm. The blue circle indicates the original position of the germanium particles. When the laser is switched on, the germanium particle is dragged up and migrated upward the laser heating spot as a result of the laser-induced thermocapillary convection. The movements of the embedded particles are dominated by the drag force along the convection flowline.

**Fig. 2:**
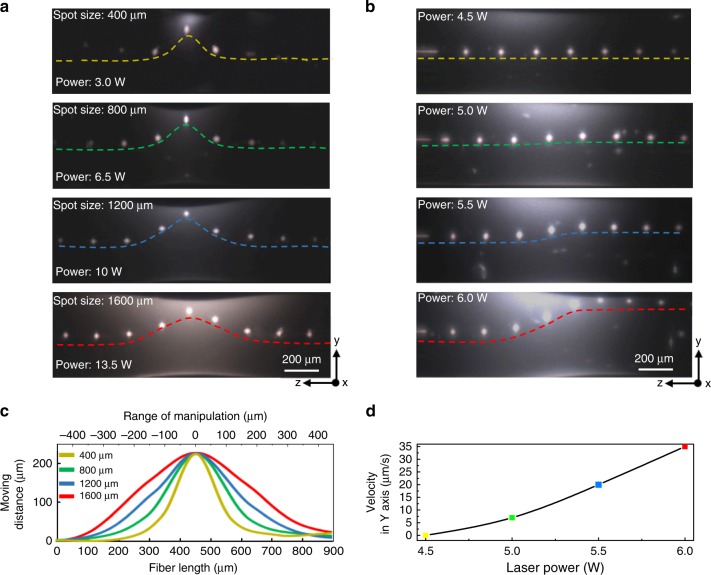
Characterisation of the laser induced thermocapillary convection and particle manipulation. **a** Laser spot size-controlled radius of thermocapillary convections for single and multiple particles migration. By increasing the laser spot size from 400 to1600 μm with laser power from 3 W to 13.5 W, the radius of the thermocapillary convection increases constantly. The range of the flow can achieve the migration of a single particle with small spot size and multiple particles with a large spot size. **b** Particles migration velocity profiles with different laser powers. The silica fibres are embedded with germanium particles with the diameter of 16 μm and distribution period of 95 μm. Fibres are fed into a CO_2_ laser beam at 50 μm s^−1^. The migration velocity increases with the increased laser power, and the germanium particle lines are relocated at different positions according to the different trapping velocities. In **a** and **b**, the laser is focused at the centre of the top surface. **c** Variation of the thermocapillary convention’s range with different laser spot sizes. **d** Variation of particles migration velocity as a function of laser power

To develop a precise method from this phenomenon for the in-fibre particles manipulation, we repeat the experiment by changing the laser power and beam spot size to investigate the controllability of the manipulations in terms of three key parameters for particles’ manipulation: the number of particles being moved, the particles migration velocity and the moving direction. First, as shown in Fig. 2a, the number of moved particles is relevant to the laser spot sizes and laser powers, which directly determine the range of the thermocapillary convection. Smaller laser spot size shortens the liquidised cladding length and induces a limited range of thermocapillary convection, which is suitable for manipulating single particle or a small group of particles. The number of migrated particles keeps increasing with increasing the laser spot size and power because larger laser spot size results in a longer liquidised cladding length and larger range of thermocapillary convection, which involves more particles into the manipulation process. Thereby it is suitable for manipulating a group of multiple particles in one time. Second, for the particles migration velocity, which is dependent on the temperature gradient over fibre cladding and cladding material’s viscosity, is found to be increased linearly with the input power. Figure 2b shows the changeable particle migration velocities achieved with different laser powers. In the thermocapillary convection, a higher laser power reflects as a higher flow velocity of mass transfer and smaller Stokes’ drag force, also noted as the velocity of particles migration. To measure the migration velocity, we feed the fibres with stable periodical particles into a laser beam with a fibre feed-in velocity (*v*_f_) of 50 μm s^−1^ and a laser spot size of 500 μm. Under the control of the laser beam, the particle chains could be dragged away from the fibre centre and relocate at the selected position among the fibre. By changing the laser power from 4 to 20 W, we can adjust the particle vertical migration velocity from 0 to 35 μm s^−1^ (See Supplementary Note 5 and Supplementary Fig. 3 for detail). Last, due to the temperature gradient within the thermocapillary convention, particles are always trapped in the heat field and moved towards the heating spot, therefore the migration direction can be controlled by changing the heating spot’s position along the fibre. In a short conclusion, by modifying the laser parameters (spot size, power, heating direction, etc.), the laser induced thermocapillary convection leads to the manipulation and migration of single particle, as well as multiple particles under precisely controllable migration velocity, which provide new possibilities to achieve sophisticated in-fibre functional structures.

### Modelling of the in-fibre thermocapillary convection

**Fig. 3:**
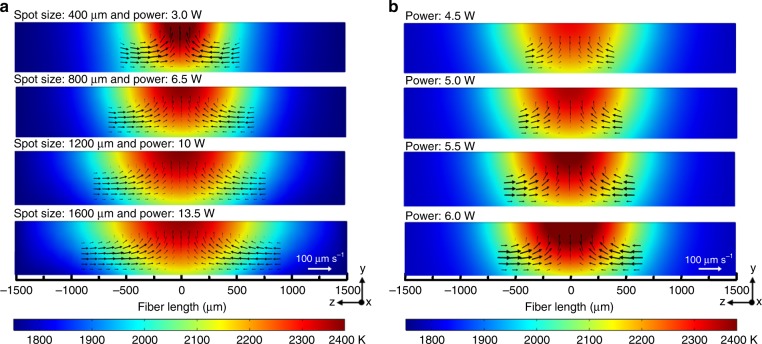
Simulation results of the laser induce in-fibre temperature gradient and flow velocity profiles of the thermocapillary convection which drags the particles up. **a** Analysis of the influence on the in-fibre temperature distribution and flow velocity profiles according to laser spot sizes. The laser spot size increases from 400 to 1600 μm with sufficient laser power from 3 W to 13.5 W, resulting in an increase of the thermocapillary flow radius. The soften length of fibre cladding also increases from 600 to 1800 μm. **b** Analysis of the in-fibre temperature distribution and flow velocity profiles according to different laser powers. The laser power increases from 4.5 to 6 W with a laser spot size of 400 μm, resulting in an increase of the flow velocity from 5 to 80 μm s^−1^. In **a** and **b**, the total fibre length is 3 mm and fibre diameter is 550 μm; The laser is focused at the centre of the top surface

To further investigate the in-fibre convection, we perform a three-dimensional axisymmetric numerical finite-element simulation model based on COMSOL Multiphysics. Since the thermocapillary convection is strongly affected by the laser’s heating effect, we combine the heat transfer and laminar flow physical modules to analyze the relationships of the migration velocity, the temperature gradient and the flow profiles^50,51^. The model aims to simulate the process of a CO_2_ laser heating a silica fibre and the accompanied thermocapillary convection. The same boundary conditions used in the experiments are applied, and the simulation parameters are shown in the supplementary information. The key to estimating the in-fibre thermocapillary convection is to determine the temperature gradient distribution, which can be calculated by the Heat Transfer Module in COMSOL^52^. Coupled with the Navier-Stokes equation, the simulation model could calculate the flow velocity field and flowlines of thermocapillary flow based on the laser induced temperature gradient. Simulated flow velocity field as a function of the temperature distribution is illustrated in Fig. 3 (Details of simulation can be found in Supplementary Note 4). For the first step, we investigate the radius of the laser-induced the thermocapillary flow. As shown in Fig. 3a, the increasing of laser beam spot size and laser power result in the fluidising of fibre with a longer length, thus the radius of the thermocapillary flow increases in the meanwhile. By adjusting the laser spot size to achieve thermocapillary convection in different ranges, we are able to control the numbers of particles to be manipulated, which is identical to the experimental findings. The minimum radius of the thermocapillary flow is smaller than the particle distribution period to involve single particle into the manipulation, determined by the minimum size of the laser spot. Obviously, increased radius will take multiple particles into the thermocapillary flow.

For the second step, we investigate the flow velocity in the convection, as well as the particles’ migration velocity using the simulation model. For the same material, temperature gradient and material viscosity are two key factors to determine the flow velocity. A higher heating temperature will lead to a larger temperature gradient and a smaller material viscosity, which both result in a more pronounced thermocapillary convection. As shown in Fig. 3b, with the increasing of laser heating power, the larger temperature gradient leads the in-fibre thermocapillary convection to become more dominant, thus the flow convection velocity keeps increasing from 5 to 80 μm s^−1^. With the flow profile and velocity, we are able to estimate the drag force on the in-fibre particles. The stress applied on the particles could be described by d*F/*d*S* *=* 1.5 *µV/R*, where *F* is the surface force, *S* is the surface area of the particle, *µ* is the material dynamic viscosity, *V* is the flow velocity and *R* is the radius of the particle^53^. The simulation model suggests that a higher laser heating power will result in a larger heating temperature gradient and a faster local flow velocity, which places stronger stress at the particle and lead to a faster particle migration velocity. Furthermore, within the same thermocapillary convection, particles with smaller radius will bear larger stress leading to faster migration velocity. In a short conclusion to the simulation study, all of these simulation results well explain and support our experimental results. Therefore, tuning the intensities and spot size of the CO_2_ laser beam allows us to precisely control the in-fibre thermocapillary flow-field and achieve an arbitrary migration of the in-fibre particles.

### In-fibre assembly of multimaterial semiconductor devices

Thermocapillary convection provides a tool to construct complex in-fibre multi-component devices, such as in-fibre p-n junction, optical resonators and optoelectronic devices. As of now, the in-fibre functional devices are mostly fabricated by capillary instability phenomenon which is coined as contact-by-breakup^15,18^. The thermal treatment induced in-fibre capillary instability phenomenon can transfer continuous fibre cores into chains of particles. As the diameter of the particle is always larger than the original cylinder core, the formation of particles with the simultaneous breakup periods can facilitate the connection of the separated fibre cores to form multiple-particle p-n molecules for semiconductor applications^54^. However, as the diameter and distribution period of the generated spheres are determined by the material’s viscosities, the diameter of fibre cores and the thermal treatment’s temperatures, very critical requirements have to be claimed on the fibre structure design, causing that only homojunction with same materials can be achieved till now^55,56^. It remains challenging on the thermal drawing process of multiple-cores structure fibre to perfect retain the fibre designed structure. Any subtle mismatching among the fibre cores will lead to unequal breakup periods and the dislocation of p- and n-type particles, failing the formation of p-n junction (as shown in Supplementary Fig. 2). More problematic, different materials always exhibit different breakup periods thus the existing contact-by-breakup method is limited in the generation of homojunctions. Indeed, the construction of in-fibre functional devices generation requires a methodology to bridge the chasm of materials and structures, and to migrate the in-fibre components. Based on the approach discussed above, the laser induced surface tension gradients within the fibre make it possible to achieve transportation and properly merging of embedded particles with different sizes and materials. Since the laser induced thermocapillary convection is symmetric around the heating point, all particles being involved in the dynamic flow are collected into the flow and migrated toward the same heating point. Eventually, contact occurs when originally separated particles are dragged into the same location, forming a multi-component in-fibre device.

**Fig. 4:**
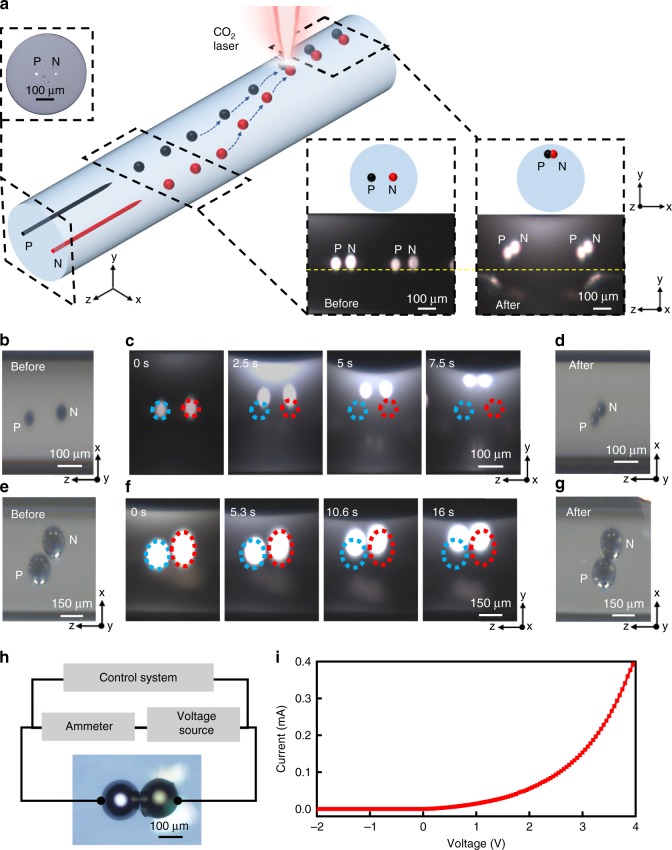
CO_2_ laser induced thermocapillary convection for structures reconstruction and p-n homojunction molecule fabrication. **a** Schematic of the asynchronous break-up of a dual-core germanium fibre and in-fibre structure reconstruction based on the CO_2_ laser induced thermocapillary convection for p-n molecules fabrication. The thermocapillary convection collects the p- and n-type spheres, moves the dislocation particles and assembly them into one p-n molecule. The insert on the top left corner shows the cross-section of the dual-core (p- and n-type) germanium fibre. The insets on the bottom show the separated p- and n-type spheres before processing and the contacted p-n junction molecule after processing. **b** Dislocated p- and n-type germanium particles with the same diameter of 40 μm. The spatial distance between two particles is around 180 μm. **c** Snapshots of in-fibre particles with a diameter of 40 μm manipulated by the CO_2_ laser power at different time-points. At the beginning (*t* = 0 s), the p- and n-type particles are located at the right and left of the laser beam. Starting from the laser switched on, the particles are dragged, migrated towards the laser beam and eventually contact with each other to form a p-n molecule (*t* = 7.5 s). **d** The p-n molecule with smaller in-fibre germanium particles is formed by the laser induced thermocapillary convection. **e** The dislocated p- and n-type germanium particles with diameters of 160 μm. The spatial distance between the particles is around 40 μm. **f** Snapshots of in-fibre particles with a diameter of 160 μm being manipulated by the CO_2_ laser power at different time-points. The moving duration is 16 s. The particles show in elliptical shape due to the convex lens effect of the cylindrical silica cladding. The actual spheres are in spherical shape as shown in **h**. **g** The p-n molecule with larger in-fibre germanium particles is formed by the laser induced thermocapillary convection. **h** Measurement diagram of the heavily doped dual-sphere germanium p-n molecule. **i** *I*–*V* characteristic of the dual-sphere germanium p-n molecule

As a proof of concept of this idea, we fabricate varisized dual-sphere p-n homo- and heterojunction particles through the proposed method of in-fibre particle manipulation. We firstly generate varisized germanium p-n homojunctions. We fabricate a dual-core fibre consisted of both p- and n-type germanium cores thereby two separated chains of spheres are formed by the laser-induced in-fibre capillary instability. Due to the different doping elements, the thermal properties of p- and n-type materials have slight differences thus resulted in the unequal core diameters, which also leads to the mismatched breakup periods of spheres (as shown in Supplementary Fig. 2). Next, to fabricate p-n junction molecules in-fibre, we apply the laser induced thermocapillary convection to achieve the manipulation and amend of the mismatching particles in one treatment. As shown in Fig. 4a, the laser heating point is located at the centre of two particles. When the CO_2_ laser is switched on, centrosymmetric dynamic convection is immediately formed and drags the pair of p- and n-type particles towards the heating point, till the two particles contacting with each other. As shown in Fig. 4, we experimentally observe that the laser induced in-fibre thermocapillary flow amends the dislocation of the p-n spheres with diameters of 40 to 160 μm and migrates them to contact, successfully forming a chain of dual-sphere p-n molecules. We have also observed that the particles migration velocity is related to particle sizes, and smaller particles experience higher migration velocities. According to the Stokes’ law, the frictional force of viscosity on a small sphere moving through a fluid is proportional to their diameters, which results in a slower migration velocity for larger particles. Furthermore, as the progress of thermocapillary convection is well controlled by the CO_2_ laser, the formed dual-sphere molecule does not merge and reshape into one sphere, avoiding the mixture between n- and p-type materials, thereby the electronic properties of both n- and p-type materials are well maintained. This statement is supported by our electrical measurement on the fabricated dual-sphere molecule. Figure 4i shows the rectifying current-voltage (*I*–*V*) characteristics of the released dual-sphere molecule, which verifies the formation of p-n junction (see methods for details).

**Fig. 5:**
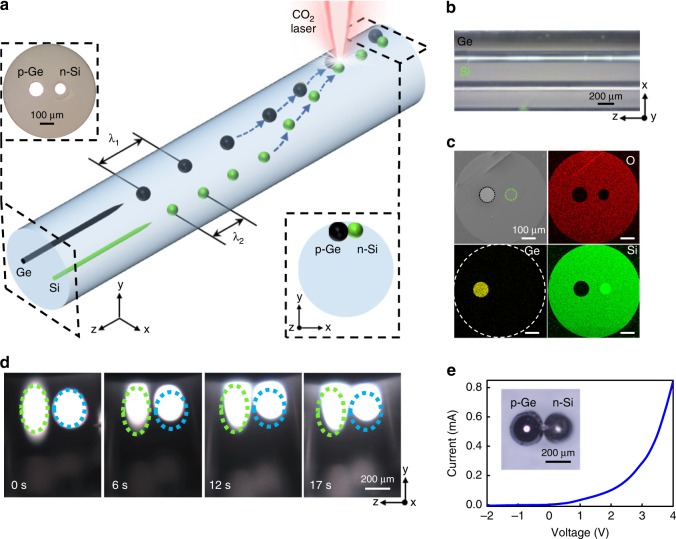
Fabrication of in-fibre p-type germanium n-type silicon heterojunction molecules based on the CO_2_ laser induced thermocapillary convection. **a** Schematic of the asynchronous break-up of a p-type germanium and n-type silicon dual-core fibre and in-fibre structure reconstruction based on the CO_2_ laser induced thermocapillary convection for p-n heterojunction molecules fabrication. Due to the different viscosities, melting points and core diameters, the periods of particle chain of germanium and silicon are different (*λ*_1_ for germanium and *λ*_2_ for silicon). **b** Microscope image of p-type germanium and n-type silicon dual-core fibre. **c** The EDS mapping of the polised cross-section of p-type germanium and n-type silicon dual-core fibre. **d** Snapshots of in-fibre Ge/Si particles manipulated by the CO_2_ laser power at different time-points. At the beginning (*t* = 0 s), the p- and n-type particles are located at the right and left of the laser beam. Starting from the laser switched on, the particles are dragged, migrated towards the laser beam and eventually contact with each other to form a p-n molecule (*t* = 17 s). The particles show in elliptical shape due to the convex lens effect of the cylindrical silica cladding. The actual spheres are in spherical shape. **e** *I*–*V* characteristic of the dual-sphere p-n germanium-silicon heterojunction molecule. The inset show the contacted pGe-nSi junction molecule after processing

In the same approach, we achieve the in-fibre heterojunction molecules with p-type germanium and n-type silicon for the first time. As the breakup period of different materials varies according to material properties such as viscosity, melting point and surface tension, it is much more challenging to achieve the in-fibre heterojunctions with the traditional method. To fabricate p-n heterojunction molecules in-fibre, we must cross the gap between materials. Using the laser induced thermocapillary convection which is universal for different materials. In this way, we finally amend the germanium and silicon particles and generate heterojunction structures in fibre. As shown in Fig. 5, we fabricate the Si/Ge dual-core fibre by transferring the two continuous cores into two chains of spheres. With the different diameter and material properties, the breakup periods of germanium and silicon are different which disables the generation of heterojunction by the conventional contact-by-breakup method. In our approach, the CO_2_ laser beam builds a centrosymmetric dynamic convection and drags a p-type Ge particle and an n-type Si particle towards the heating point, forming a heterojunction. We experimentally realised the movement of the germanium and silicon particles and generation of the in-fibre heterojunction molecules (Fig. 5d). Figure 5e demonstrates the *I*–*V* characteristics of the released Ge/Si p-n heterojunction. In short, by precisely controlling the CO_2_ laser’s treatment, the convection manipulation empowers us a new way to generate sophisticated in-fibre functional structures by joining multiple components and re-writing the particles distributions without any limitation of materials. Note, by adjusting the laser heating area, the in-fibre spherical particle generation and in-fibre functional structure formation can be achieved in one laser thermal treatment.

## Discussion

Taking advantage of the CO_2_ laser induced liquefaction approach, the in-fibre thermocapillary convection enables a universal method to accurately manipulate in-fibre particles and precisely control the reconstruction of in-fibre complex components, which can enrich the family of multifunctional fibres with a variety of materials. This size- and materials-independent approach allows us to relocate the in-fibre single or multiple particles, as well as to rewrite the in-fibre component distribution under a controlled migration velocity. To demonstrate the superiority of this approach, fibres with dual-chain of p- and n-type semiconductor particles were reconstructed. The thermocapillary convection is utilised to amend the dislocation between the p- and n-type spheres, creating the contact of the particles to form p-n dual-sphere homo- and heterojunction molecular and opening a path to achieve flexible wearable devices, large-area photodetecting devices, and imaging and diagnostic probes. It is worth noting that this approach is universal, making it also possible to develop three dimensions complex in-fibre functional structures, such as p-i-n and metal-semiconductor-metal (M–S–M), by applying programmable laser heating effects with multi-directional controls.

## Methods

### Fibre thermal drawing process

(1) Single germanium-core silica-cladding fibre: to achieve the 5 and 12 μm germanium-core silica-cladding fibres, a multiple drawing process was developed. First, we inserted a 2-mm germanium rod into a silica tube (Scott Glass Products) with a core/cladding structure shown in Supplementary Fig. 1. The preform was settled into fibre tower and was drawn down to the germanium-core silica-cladding fibre at 1950 °C. The resulted fibre was inserted into a new silica tube and is redrawn at 2100 °C into the germanium-core fibre with the core diameter of 5 and 12 μm. The preform fed-in speeds and the fibre drawing speeds could be found in the supplementary information. (2) Dual p- and n-cores germanium/silica fibre: The process started with the preparation of fibre preforms with dual cores (P and N types). For the large dual-core fibre, we inserted a heavily doped p-type germanium rod (electron acceptors concentration *N*_A_ > 10^18^ cm^−3^) and an n-type germanium rod (electron donor concentration *N*_D_ > 10^19^ cm^−3^) with the diameter of 1 mm into a silica tube. The gap was filled by varisized silica rod. For the small dual-core fibre, we inserted p-type germanium fibre and n-type germanium fibre with the core diameters of 0.7 mm into a silica tube. This preform also was settled in fibre drawing tower and were drawn into micron fibre at 1950 °C. (3) Dual p-Ge and n-Si cores silica-cladding fibre: the preparation of fibre preforms with p-Ge and n-Si cores is same with dual p- and n- cores germanium/silica fibre. We inserted a heavily doped n-type silicon rod (electron acceptors concentration *N*_A_ > 10^19^ cm^−3^, diameter *D* = 2 mm) and an p-type germanium rod (electron donor concentration *N*_D_ > 10^18^ cm^−3^, diameter *D* = 3 mm) into a silica tube. We filled the interspace by varisized silica rod. This preform also was settled in fibre drawing tower and was drawn into micron fibre at 1960 °C.

### In-fibre spherical particles generation

The method to generate in-fibre functional particles has been reported in our previous publication^25^. CO_2_ laser spot heats the selected fibre zone to melt the germanium functional core and soften the silica cladding. For the reason of the surface tension at the core/cladding interface, the continuous germanium fibre core breaks into a chain of spheres with the same diameter and distribution period. The generated in-fibre spheres line up at the same place as the fibre core. In detail, the germanium-core silica-cladding fibre was fed into a laser heating beam with a fibre feed-in velocity (*v*_f_) of 50 μm s^−1^. The laser power was 3.5 W and the laser spot size focused on the fibre was 500 μm. As the temperature increasing, the silica cladding was softened, and the germanium was molten too. To reduce the surface tension at the interface of germanium and silica, a sinusoidal perturbation occurred at the surface of the germanium fibre core cylinder. With the development of the perturbation, the continuous fibre cylinder core broke up into a chain of spheres. For the dual pair spheres, the resulted dual-cores fibres were also fed into the laser beam (see more details in Supplementary Note 3). For the small dual-cores fibre, the laser power was 3.5 W, the laser spot size on fibre was 500 μm the fibre feed-in velocity (*v*_f_) was 10 μm s^−1^. For the large dual-core fibre, the laser power was 6 W, the laser spot size on fibre was 1500 μm and the fibre feed-in velocity (*v*_f_) was 10 µm s^−1^ to avoid the cracks induced by the built-in stress. The laser beam was focused on the centre of the dual cores to balance the heating temperature.

### Devices of laser-induced in-fibre thermocapillary effect

In this experiment, we used a CO_2_ laser (Diamond C20) with a wavelength of 10,600 nm and the maximum laser power is 20 W. The laser spot size applied on the fibre surface could be adjusted from 500 to 2000 μm by a customised lifting platform. The fibre was fixed in a group of linear transformation stages (Thorlabs LNR50S/M). The fibre could be rotated during the heating process with the fibre rotators (Newport 466 A). The particle manipulation processes were recorded by a CMOS camera (Thorlabs DCC1545M) with a 5X focusing microscope objective lens.

### In-fibre pn homo- and heterojunction generation

For the 40-μm p- and n-type homojunction germanium spheres group, the laser beam was focused on the top surface of the fibre cladding at the centre of the bi-spheres. The laser spot size on the fibre was 500 μm and the laser power was 7.5 W. For the 160 μm p- and n-type homojunction germanium spheres group, the laser beam was also focused on the top surface of the fibre cladding at the centre of the bi-spheres. The laser spot size on the fibre was 800 μm and the laser power was 11 W. For the n-type germanium and p-type silicon heterojunction spheres groups, the laser beam was focused on the top surface of the silica cladding. The laser spot size on the fibre was 800 μm and the laser power was 11 W.

### Measurement of current–voltage of the generated pn junction

The bi-spherical homo-and heterojunction particles consisted of heavily doped N and P-type spheres were released from the silica cladding in 49% hydrofluoric acid solution (Sigma Aldrich, Singapore). We used conductive silver adhesives to achieve the electrode connections. A Keithley 4200A-SCS was used to apply the voltage between the bi-spherical molecule and to measure the current.

## Supplementary information

Supplementary Information

## Data Availability

All relevant data supporting the finding of this study are available within this article, its Supplementary Information or from the corresponding author upon reasonable request.
