# Supplementary material for: In-fibre particle manipulation and device assembly via laser induced thermocapillary convection
Source: Nat Commun. 2019 Nov 15;10:5206. doi: 10.1038/s41467-019-13207-0 (PMC6858441; doi:10.1038/s41467-019-13207-0)
Supplement: Supplementary file 1 — Supplementary Information [file 41467_2019_13207_MOESM1_ESM.pdf]

In-Fibre Particle Manipulation and Device Assembly *via* Laser Induced Thermocapillary Convection

Zhang et al.

Supplementary Note 1

Dominant Mechanism

We propose a novel laser-based approach that is able to precisely manipulate in-fibre micro-sized particles for complex structure fabrication. To migrate the in-fibre particles, we use CO_2 laser to fluidize part of fibre cladding, creating thermocapillary convection. Since the existing of Gaussian laser beam, conductive heat transfer, and the heat-exchange with the ambient environment, the temperature distribution among the heated fibre presents as a Gaussian distribution, which results to a temperature gradient spreading in three dimensions, as well as a gradient of temperature-depended density and surface tension along the fibre. The temperature gradient introduces flow convection which applies drag stress onto in-fibre particles. There are two temperature-related phenomena that drive the convection: natural convection and Marangoni convection. The natural convection is caused by the Archimedes lift force induced by the temperature-depended density gradient of cladding materials. The Marangoni convection is driven by the forces induced by the temperature-depended surface tension gradient. In order to analyze the laser induced in-fibre convection and develop an estimation tool of the convection drag force and trapping velocity, we use microfluidic dimensionless analysis to study the phenomenon.

First, the onset of the natural convection can be determined by the Rayleigh Number (Ra):

$$Ra = \frac{\beta \Delta T g L^3}{\alpha \mu} \qquad \text{Eq. 1}$$

Where β is the volume thermal expansion coefficient, ΔT is the difference in temperature, g is the gravitation acceleration, L is the liquid layer thickness of the convection, α is the thermal diffusivity, and μ is the dynamic viscosity of liquid. Second, the thermocapillary convection, also known as the thermal Marangoni convection is generated by the Buoyant force, surface tension

force as well as the viscous force. The relationship between the surface tension and viscous force can be described as the Marangoni number (*Ma*):

$$Ma = -\frac{d\sigma}{dT}\frac{L\Delta T}{\mu\alpha} \qquad \text{Eq. 2}$$

where dσ/dT is the temperature coefficient of the surface tension, L is the thickness of liquid layer, ΔT is the temperature difference, μ is the dynamic viscosity of cladding materials [1] and α is the thermal diffusivity[2,3]. And the relationship between the Buoyant force and viscous force can be described as the Rayleigh as shown in Eq. 1. Therefore, the ration between Ra and Ma gives the dynamic Bond Number (*Bo*)

$$Bo = \frac{Ra}{Ma} = \frac{\beta g L^2}{\frac{d\sigma}{dT}} \qquad \text{Eq. 3}$$

To calculate these dimensionless numbers, the physical properties of fused silica in table 1 are used and *Bo* is around 10^{-4}. So, the Marangoni effect is the dominant mechanism and the natural convection is negligible.

Supplementary Table 1: Physical properties of fused silica

Property	Parameter	Value	Ref
Dynamic Viscosity	μ	~10^3 Pa S	4
Temperature Coefficient of Surface Tension	$\frac{d\sigma}{dT}$	3×10^{-5} N (m K)$^{-1}$	5
Density	ρ	2.03 g cm^{-3}	6
Thermal Diffusivity	α	~10^{-6} m^2 s^{-1}	7
Volumetric Thermal Expansion Coefficient	β	~10^{-5} K^{-1}	8

The velocity of the thermocapillary flow

As the thermocapillary convection is the dominant mechanism, the convection flow's velocity can be estimated as $V = d\sigma / \mu$, where $d\sigma$ is the temperature-depended difference of the surface tension[9]. As the temperature is around 2500 K to 2800 K and ΔT is around 150 K to 200 K, the viscosity of silica at this temperature is around 10^2 to 10^3 Pa S. Therefore, the estimated flow velocity is around 5 to 100 μm s^{-1}, which agrees with the COMSOL simulation results.

Supplementary Note 2

Preform preparation and fibre fabrication

Before the fibre thermal drawing process, we need to prepare preforms for the single core and the dual core (n- and p-type) semiconductor fibres. In order to compose a preform, there are several general rules to be followed: (1) the cladding material should be amorphous, (2) the molten/soften temperatures of cladding and core materials should be comparable, and (3) the thermal expansion coefficients of all the materials should be in the same interval[10,11]. As we use germanium as the semiconductor functional material, silica is suitable to be the cladding materials [1,12].

For the single core fibre, a rod of functional materials is inserted into a silica tube and the ports of the tube are vacuum sealed to prevent oxidation or decomposition of functional materials under the high temperature (**Supplementary Figure 1a**). The generated fibres can be inserted into a new tube and redrawn several times to achieve a fibre with smaller core diameters. For the dual core fibre, two rods with p- and n-types materials are inserted into one silica tube. Also, the interspace is filed by the silica rods. Same as the single one, the ports of the preform are also vacuum sealed (**Supplementary Figure 1b**). It's worth noting that the heavily doped n- and p-type germanium

rods have distinguishing thermal properties with the intrinsic material, such as the molten point and the dynamic viscosity. Their properties' differences will influence the structure of resulting fibres.

Then the preform is placed into a fibre drawing tower and thermally drawn into micron fibres. The diameters of the resulting fibre could be controlled by the preform feed-down speed and the thermally drawing speed. The parameters of the thermal drawing process are covered in this article and shown in table 2.

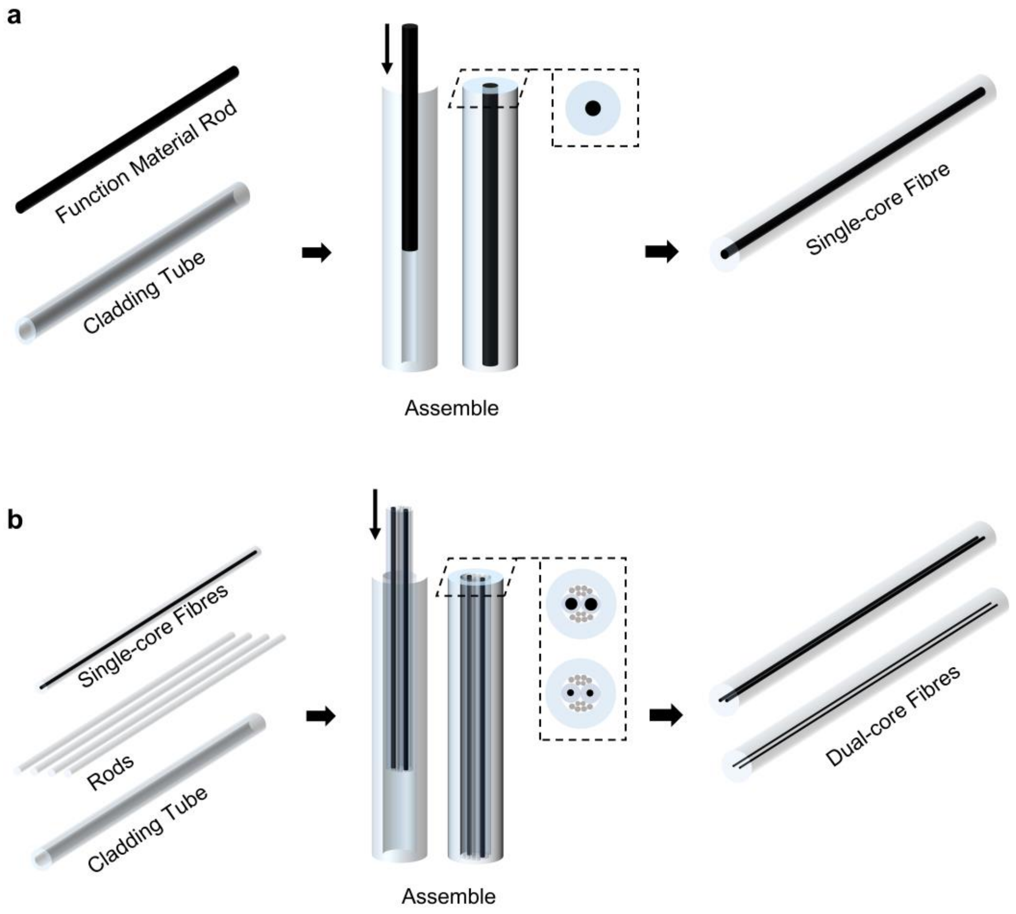

Supplementary Figure 1: Preform preparation process for multimaterial fibre fabrication. a) Single-core multimaterial fibre preform preparation. b) Dual-core multimaterial fibre preform preparation.

Supplementary Table 2: Parameters used in fibre thermal drawing process

Fibre type	Fibre Materials	Preform outer/inner diameter (mm)	Fibre outer/inner diameter (μm)	Drawing temperature (°C)	Preform feed velocity (mm min^{-1})	Drawing velocity (m min^{-1})
Single core fibre	Silica/Ge	10 / 0.11	500 / 5	1950	5	2
Single core fibre	Silica/Ge	4 / 0.11	500 / 12	1950	5	0.32
Dual core fibre	Silica/(p-Ge + n-Ge)	12 / (1:1)	550 / (45+45)	1960	2	0.95
Dual core fibre	Silica/(p-Ge + n-Ge)	4 / (0.07:0.07)	550 / (12+12)	1950	9	0.48
Dual core fibre	Silica/(p-Ge + n-Si)	20 / (3:2)	650 / (100+67)	1960	0.75	0.70

Supplementary Note 3

PN Sphere Pairs Fabrication and Period Dislocation

To fabricate the in-fibre pn homo- and heterojunction molecule, we need to fabricate the p- and n-pair semiconductor spheres firstly. With the dual core semiconductor fibres, we can use a CO_2 laser to selectively heat a part of the fibre which induces capillary instability into the interface between the core and cladding materials. The instability results in breaking-up of the continuous dual cores. However, the heavily doped p- and n-type germanium materials, as well as the p-type germanium and n-type silicon have distinguished thermal properties. According to Tomotika's analysis[13,14], the breakup period of the capillary instability is decided by the material viscosity and interfacial surface tension. Thus, the breakup periods of the p- and n-type cylindrical cores are different, which leads to the dislocation between the p- and n-type spheres in the z-direction (**Supplementary Figure 2**). Moreover, the existing volume change together with the distinguishing thermal expansion coefficients between the core and cladding materials induces

gigapascal-level built-in stress onto the semiconductor fibre cores that may break the cladding during the fibre thermal drawing process. To avoid the mixture of the p- and n-type materials, we design a gap between the p- and n-type cores to maintain the fibre structure. But the necessary large gap leads to the unwanted dislocation of the dual sphere in x-direction. Therefore, the generated spheres cannot connect to form functional pn molecules (as shown in **Supplementary Figure 2**). The asynchronized breakup of the in-fibre particles is an unavoidable common phenomenon. Therefore, the laser induced in-fibre components migration is indispensable for in-fibre complex functional structure fabrications.

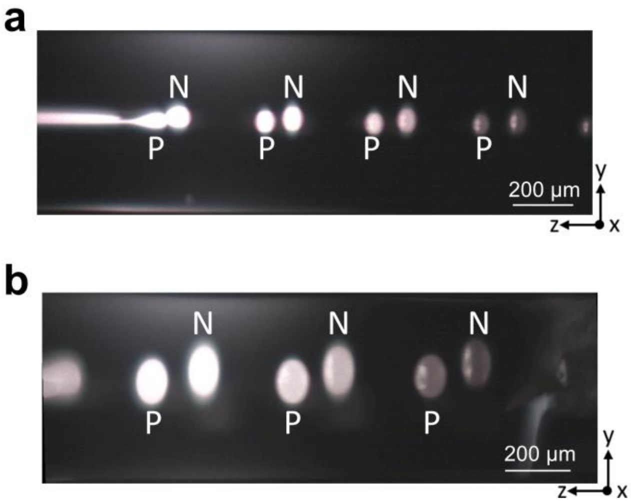

Supplementary Figure 2: PN sphere pairs fabrication and period dislocation. a) Generation of germanium p and n spherical paritcles with diameters of 40 μm. b) Generation germanium p and n spherical paritcles of with diameters of 160 μm.

Supplementary Note 4

Analysis and Calculation of Laser Induced Thermal Treatment and In-Fibre Marangoni convection

To better understand the laser induced in-fibre thermocapillary convection, we use a three-dimensional numerical finite element method simulation by the software COMSOL for a model of the CO_2 laser induced in-fibre thermal heating field, as well as the temperature-depended convection flow field. Based on the Heat Transfer Module, the laser induced thermal field can be described by the energy balance equation balance $\rho C_p \frac{\partial T}{\partial t} + \rho C_p u \cdot \nabla T = \nabla \cdot (k \nabla T) + Q$, where ρ is the local density of the materials; C_p is the specific heat capacity; T is the local temperature; u is the velocity of materials; and Q is the heating power per unit volume[15]. The energy distribution of laser heating spot is in Gaussian. The model describes the temperature distribution along the fibre as a function of the laser spot size and laser power. When the laser induced thermal heating, temperature is higher than the silica glass transition temperature, the silica cladding will transfer into liquid phase and the temperature-depended surface tension will change accordingly. The unevenly distributed surface tension leads to the flow currents. By combining with Laminar Flow Module, we can simulate the thermocapillary flow currents based on the Navier-Stokes equation. The intensity of the thermocapillary convection can be described by the dimensionless Marangoni number (Ma). The Ma number increases when the intensity of the convection increasing[2,3]. According to the thermal properties of the fused silica, the increasing heating temperature should enhance the in-fibre convections in higher flow speed and larger convection ranges. Simulation model verifies the proposed theory, as well as the experimental results.

Supplementary Note 5

Measurement of Migration velocity

Measurement of migration velocity is based on image processing of a series of CCD camera images. Depending on the different application scenarios, the in-fibre particle manipulation can be achieved both in a static fibre and a moving fibre.

a. For a static fibre (as shown in Figure 2a), the measurement of the migration velocity (v_m)is calculated by the particle displacement distance (*d*) and the time (*t*) it takes

$$v_m = \frac{d}{t} \tag{Eq. 4}$$

b. For a moving fibre (as shown in Figure 2b), the measurement of the velocity is calculated by the fibre moving velocity (v_f) and the particle displace distance (Δz), as shown in Supplementary Figure 3.

$$t = \frac{\Delta z}{v_f} \tag{Eq. 5}$$

$$v_m = \frac{\Delta y \cdot v_f}{\Delta z} \tag{Eq. 6}$$

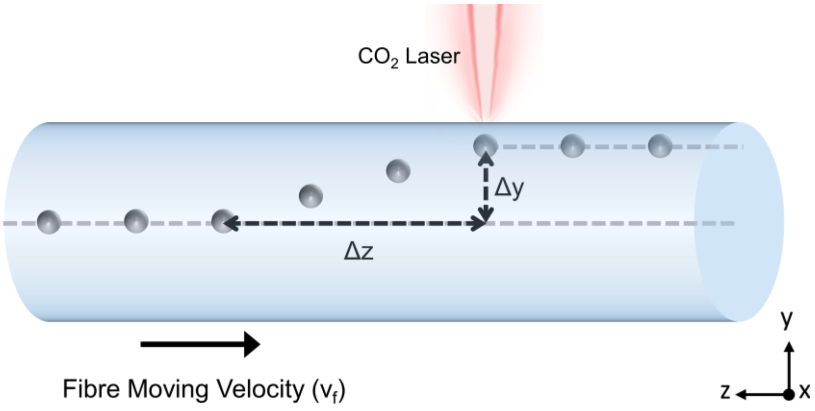

Supplementary Figure 3: Schematic of the in-fibre particle migration velocity calculation.

Supplementary References:

1 Bacon, J. F. & Hasapis, A. A. Viscosity of molten silica. *Journal of Applied Physics* **30**, 1470-1471 (1959).

2 Block, M. J. Surface tension as the cause of Bénard cells and surface deformation in a liquid film. *Nature* **178**, 650 (1956).

3 Joseph, D. D. *Stability of fluid motions I*. Vol. 27 (Springer Science & Business Media, 2013).

4 Doremus, R. H. Viscosity of silica. *Journal of Applied Physics* **92**, 7619-7629 (2002).

5 Parikh, N. Effect of atmosphere on surface tension of glass. *Journal of the American Ceramic Society* **41**, 18-22 (1958).

6 Aksay, I. A., Pask, J. A. & Davis, R. F. Densities of SiO_2-Al_2O_3 melts. *Journal of the American Ceramic Society* **62**, 332-336 (1979).

7 Sergeev, O., Shashkov, A. & Umanskii, A. Thermophysical properties of quartz glass. *Journal of Engineering Physics* **43**, 1375-1383 (1982).

8 Souder, W. H. & Hidnert, P. *Measurements on the Thermal Expansion of Fused Silica, by Wilmer Souder, Peter Hidnert*. (US Government Printing Office, 1926).

9 Protasov, C., Khmyrov, R., Grigoriev, S. & Gusarov, A. Selective laser melting of fused silica: interdependent heat transfer and powder consolidation. *International Journal of Heat and Mass Transfer* **104**, 665-674 (2017).

10 Tao, G., Stolyarov, A. M. & Abouraddy, A. F. Multimaterial fibers. *International Journal of Applied Glass Science* **3**, 349-368 (2012).

11 Sorin, F. *et al.* Multimaterial photodetecting fibers: a geometric and structural study. *Advanced Materials* **19**, 3872-3877 (2007).

12 Sato, Y. *et al.* Viscosity and density of molten germanium. *High Temperatures-High Pressures* **32**, 253-260 (2000).

13 Kinoshita, C., Teng, H. & Masutani, S. A study of the instability of liquid jets and comparison with Tomotika's analysis. *International Journal of Multiphase Flow* **20**, 523-533 (1994).

14 Tomotika, S. On the instability of a cylindrical thread of a viscous liquid surrounded by another viscous fluid. *Proceedings of the Royal Society of London. Series A-Mathematical and Physical Sciences* **150**, 322-337 (1935).

15 Darif, M., Semmar, N. & Orléans Cedex, F. in *Proceedings of the COMSOL Conference 2008 Hannover.*
